# Collision of a Giant histiocytic sarcoma with a basal cell carcinoma: causality or coincidence?^☆^

**DOI:** 10.1016/j.abd.2021.02.015

**Published:** 2022-11-11

**Authors:** Inés Gracia-Darder, Julián Boix-Vilanova, Cristina Gómez Bellvert, Luis Javier Del Pozo Hernando

**Affiliations:** a Department of Dermatology, Son Espases University Hospital, Palma de Mallorca, Balearic Islands, Spain; b Department of Pathology, Son Espases University Hospital, Palma de Mallorca, Balearic Islands, Spain

Dear Editor,

An 85-year-old woman presented with a 6-month-old tumor in her back that grew to reach 15 cm in diameter. It was not accompanied by constitutional symptoms or cytopenias. The lesion was exophytic, multinodular, with reddish and white-yellowish areas (Fig. 1). At the upper pole of the tumor, there was a flatter milky red area with regression areas and ovoid nests on dermoscopy, which was histologically confirmed as a basal cell carcinoma (BCC) (Fig. 2 a‒c). A biopsy of the most voluminous tumor (Fig. 3a) showed dermal sheets of atypical pleomorphic cells. Tumor cells stained positively for CD68, CD163, CD45, lysozyme (Fig. 3 b‒d), and vimentin, and were negative for lymphoid, dendritic, epithelial, and melanocytic markers. Fine needle aspiration of a palpable left axillary mass showed histological findings similar to those described before (Fig. 1). Based on these findings, the diagnosis of cutaneous histiocytic sarcoma (HS) was made. Computed tomography showed multiple axillaries and retropectoral lymphadenopathies accompanied by millimeter nodules in the liver and lungs. The patient received palliative treatment and died after three months.

**Figure 1:**
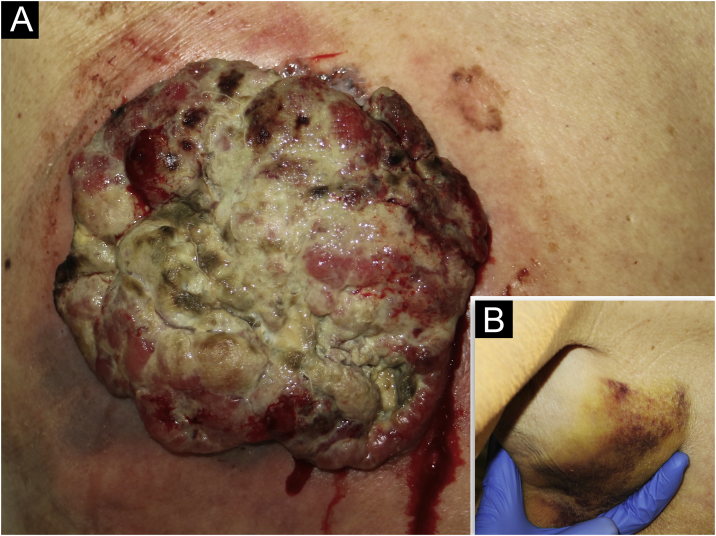
(a) Exophytic multinodular tumor of 15 cm in diameter with a festooned edge, white-yellowish with reddish hemorrhagic areas, surrounded by an asymmetrical erythematous halo. (b) Subcutaneous palpable mass, tough, of 5 cm diameter located in left axillae.

**Figure 2:**
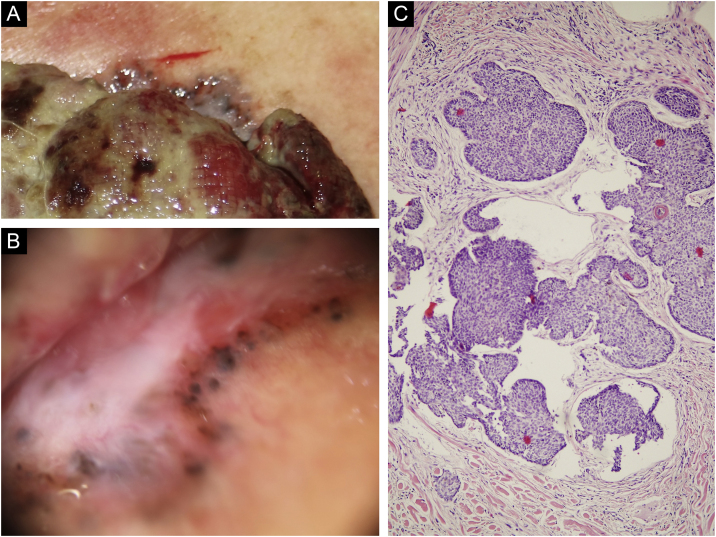
(a) On the upper pole of the giant mass can be observed a greyish bright area different from the rest of the periphery. (b) Dermoscopy of the upper pole revealing a flatter milky red area with whitish regression areas and ovoid nests. (c) Light microscopy with basaloid cells with scant cytoplasm and elongated hyperchromatic nuclei, peripheral palisading and peritumoral clefting, (Hematoxylin & eosin, ×100).

**Figure 3:**
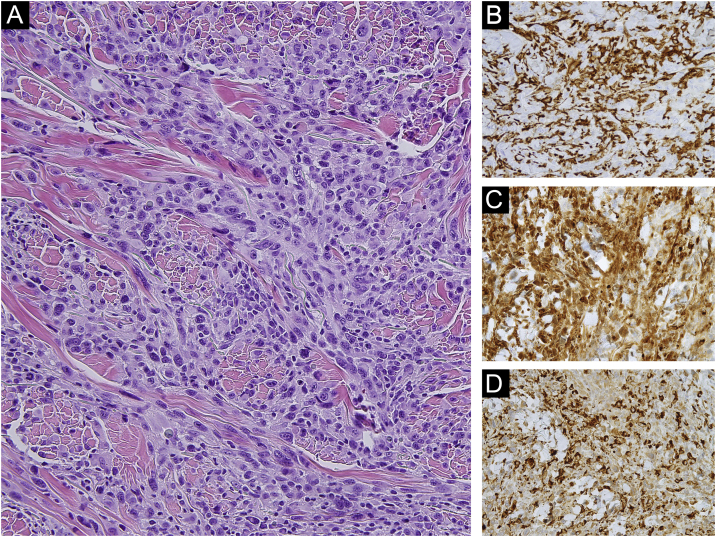
(a) Sheets of atypical and pleomorphic cells with numerous mitoses and necrotic areas. The tumor cells have prominent nucleolus, excentric cytoplasm with a nucleous/cytoplasm disproportion. Tumor cells stained positively for CD163 (×400) (b), Lisozime (×400) (c), and CD68 (×400) (d).

Histiocytic sarcoma is a rare hematologic neoplasm of histiocytic or dendritic origin, with only a few hundred cases reported. It can be seen at any age and appears to have a slight male predominance.^1^, ^2^ HS commonly presents as a painless solitary mass in an extranodal location (gastrointestinal tract, soft tissue, skin, spleen, or liver). Skin is affected in almost 7% of the cases, with lesions described predominantly as asymptomatic unique plaque and or nodule.^1^, ^2^ Dermoscopy has been described as homogeneous yellow background with whitish linear stripes, and arborizing vessels on the periphery.^3^ Primary cutaneous HS usually presents as a localized disease, with a better prognosis than extracutaneous HS, mainly because it benefits from early treatment.^1^ In contrast, extracutaneous HS has a mortality rate of 58% with a limited response to chemotherapy.^4^ In our case, given the poor evolution of our patient, we interpret cutaneous involvement as a result of contiguous extension from the axillary mass, and not as primary cutaneous HS, although both types would be histologically indistinguishable. HS can be associated, with other hematologic malignancies, suggesting that B cells can be transdifferentiated to a malignant histiocyte.^1^, ^2^ The collision of an HS with a BCC has not been previously described in the literature. In our case, although it could be coincidental, the presence of a previous BCC in the area could have influenced the HS location. In this sense, three cases of “*de novo*” Langerhans cell histiocytosis and one case of indeterminate cell histiocytosis located in sites previously occupied by a BCC have been described. BCCs can create a cytokine environment that promotes cellular hyperplasia but also facilitates the recruitment of latent HS.^5^ Additionally, HS could have induced the development of a BCC at the site of its contiguous skin involvement, but this is unlikely due to the low growth rate of the BCC, compared to HS. Given the rarity of skin involvement in HS, it is necessary to collect more cases to obtain a detailed understanding of its biological behavior.

## Financial support

None declared.

## Authors' contributions

Inés Gracia-Darder: Approval of the final version of the manuscript; critical literature review; data collection, analysis, and interpretation; effective participation in research orientation; intellectual participation in propaedeutic and/or therapeutic; management of studied cases; manuscript critical review; preparation and writing of the manuscript; statistical analysis; study conception and planning.

Julián Boix-Vilanova: Approval of the final version of the manuscript; data collection, analysis, and interpretation; effective participation in research orientation; intellectual participation in propaedeutic and/or therapeutic; management of studied cases; manuscript critical review.

Cristina Gómez Bellvert: Approval of the final version of the manuscript; data collection, analysis, and interpretation; effective participation in research orientation; manuscript critical review.

Luis Javier Del Pozo Hernando: Approval of the final version of the manuscript; critical literature review; effective participation in research orientation; intellectual participation in propaedeutic and/or therapeutic; management of studied cases; manuscript critical review.

## Conflicts of interest

None declared.
